# Revolutionizing Gluteal Augmentation With the “Round Ass Technique”: A Polymethylmethacrylate-Based Approach

**DOI:** 10.7759/cureus.105721

**Published:** 2026-03-23

**Authors:** Hamilton Couto, Jessica Mesquita

**Affiliations:** 1 Department of Dermatology, Clinica Venusta, São Paulo, BRA

**Keywords:** body contouring, gluteal augmentation, injectable fillers, polymethylmethacrylate, polymethylmethacrylate (pmma)

## Abstract

Polymethylmethacrylate (PMMA) gluteal augmentation has demonstrated favorable safety, efficacy, and cost-effectiveness when performed by experienced professionals. However, until 2023, available evidence remained limited by methodological bias and low levels of scientific rigor. A recent study has suggested that PMMA use for gluteal augmentation may be safe and effective, reporting a low rate of adverse events in a large retrospective patient sample.

This technical report describes the “Round Ass Technique,” a PMMA-based gluteal augmentation approach developed to correct substantial volume deficiencies and enhance aesthetic outcomes in patients dissatisfied with prior treatments. The procedure was performed on a 28-year-old female patient who received detailed counseling regarding potential risks, alternative interventions, and the permanent nature of PMMA before providing informed consent. Biossimetric® PMMA was injected according to a standardized protocol by qualified professionals. No intraoperative or postoperative complications were observed. Long-term follow-up exceeding two years demonstrated consistent patient satisfaction and confirmed the safety, durability, and aesthetic stability of the “Round Ass Technique.” This is, to the best of our knowledge, the first standardized approach for gluteal augmentation using PMMA.

## Introduction

According to the 2022 statistical report by the American Society of Plastic Surgeons, neuromodulator injections were the leading minimally invasive procedure, totaling 8.7 million treatments. Dermal fillers followed closely and are used to address aging, volume loss, scars, cellulite, and body contouring [1]. Fillers are categorized as nonpermanent, semipermanent, and permanent; their selection is based on composition, indication, and efficacy [2]. Safe and effective application demands experience, and the ideal filler should offer long-lasting aesthetic results, biocompatibility, stability, and low complication rates [3].

Polymethylmethacrylate (PMMA)-collagen gel is among the most studied fillers. FDA trials involving a five-year follow-up of 1,008 participants showed that 83% reported satisfaction at five years [4]. In Brazil, the Agência Nacional de Vigilância Sanitária (ANVISA) approved polymethylmethacrylate (PMMA) gel for treating lipodystrophy in patients with acquired immunodeficiency syndrome (AIDS) and for facial/body volumetric correction [5]. Biossimetric® is the authorized product, classified as a class IV (maximum risk) health product and requiring administration exclusively by qualified professionals [5].

There is a growing demand for gluteal augmentation, driven by aesthetic ideals that vary across cultures and genders. A recent study identified the ideal waist-to-hip ratio as 0.75 in women and 0.85 in men, with preferences for buttock shape differing by gender and viewing angle [6]. While surgical options such as the Brazilian butt lift (BBL) and gluteal implants remain popular, they are also associated with high complication rates [7-9].

This has led to increased interest in minimally invasive approaches using fillers such as hyaluronic acid and poly-L-lactic acid; however, the results are often temporary and provide limited satisfaction [10,11]. In contrast, PMMA-based fillers, which are well-established in facial aesthetics and more recently in body aesthetics, offer permanent outcomes and a favorable safety profile for gluteal augmentation [12,13]. However, no standardized techniques using PMMA for this application have been described in the literature.

This study therefore describes our “Round Ass Technique,” a PMMA-based gluteal augmentation method, and reviews the relevant literature regarding its indications, safety, and complications.

## Technical report

The “Round Ass Technique” was performed on a young patient with no other comorbidities but with significant gluteal volume defects and low self-esteem due to unsatisfactory aesthetic outcomes from prior treatments. Our 28-year-old female patient was informed of potential risks, alternative options, and the permanent nature of PMMA. After providing informed consent, she opted for this technique, which was performed in August 2023.

Biossimetric® PMMA was injected following our standardized protocol at Clinica Venusta. No complications were observed during or after the procedure. Long-term follow-up (>2 years) showed sustained satisfaction and confirmed the safety and stability of the treatment. Patient satisfaction was assessed using a 1-10 numerical scale and was consistently rated 10 at one-month, three-month, six-month, one-year, and two-year follow-ups.

Technique description

Pre-procedure

The procedure requires the following materials: sterile gloves, a procedure gown, sterile drapes, aqueous chlorhexidine, a sterile surgical marker, gauze, and a local anesthetic composed of 100 mL saline mixed with 20 mL lidocaine with epinephrine. Additional supplies include 3 mL syringes containing 30% Biossimetric® PMMA, 3 mL syringes of 15% Biossimetric® PMMA, 20 mL and 5 mL syringes, a semiflexible cannula (18G/100 mm), a 30 G yellow needle, an 18 G pink needle, and a surgical chlorhexidine brush.

Before the procedure, informed consent must be obtained and documented. The patient should then be assessed in standing (backward, 45°, and 90°), seated, and prone positions. Standardized pre-procedure photographs and videos are taken using a black background, a tripod, proper lighting, and a high-resolution camera, ensuring relaxed muscles and appropriate underwear. With the patient in the prone position, anatomical markings are made. Thirty minutes before the procedure, administer 1 g intramuscular ceftriaxone and 100 mg intramuscular ketoprofen. The gluteal region is then disinfected with gauze and chlorhexidine. Finally, the surgical team performs handwashing with a chlorhexidine brush and dons a sterile gown and gloves before beginning the procedure.

Marking

With the patient in the prone position, the sacrococcygeal joint is first palpated. Using a surgical marker, a horizontal line is drawn from this point toward the greater trochanter, dividing the buttock into two equal halves. At the midline level of each thigh, a vertical line is then drawn perpendicular to the horizontal one, dividing each buttock into four quadrants: superomedial (SM), superolateral (SL), inferomedial (IM), and inferolateral (IL). The intersection of these lines defines the central primary access point (CPAP). The lateral primary access point (LPAP) is determined according to the patient’s height: For patients under 1.60 m, from the CPAP, move 1 cm upward and 2 cm laterally; for those between 1.60 m and 1.70 m, move 2 cm upward and 3 cm laterally; and for those taller than 1.70 m, move 3 cm upward and 3 cm laterally. The same markings are repeated on the opposite buttock.

To define the medial primary access point (MPAP), palpate laterally from the sacrococcygeal tip along the horizontal line toward the left until reaching the edge of the muscle at its coccygeal origin, and then, move 1 cm laterally; this point marks the MPAP. The process is repeated on the right side. At this stage, three primary access points, CPAP, LPAP, and MPAP, are established. The inferolateral quadrant is accessed through all three, while the remaining quadrants utilize only CPAP and LPAP. CPAP and LPAP serve as universal points, whereas MPAP is specific to the inferolateral quadrant, ensuring a homogeneous and anatomically guided product distribution.

Secondary access points are then identified as follows: the femoral secondary access point (FSAP) is located by extending the inferior gluteal fold laterally and drawing a bisector within the inferolateral quadrant, marking the intersection as FSAP (repeated bilaterally). From FSAP, a vertical line parallel to the central line is drawn; its intersection with the horizontal line defines the trochanteric secondary access point (TSAP), which may also be located by palpating the greater trochanter and tracing upward along the muscle edge. On the same vertical line, the intersection with the bisector of the superolateral quadrant marks the iliac secondary access point (ISAP). In the superomedial quadrant, where the bisector meets the medial border of the gluteus maximus origin on the sacrum, the sacral secondary access point (SSAP) is identified.

**Figure 1:**
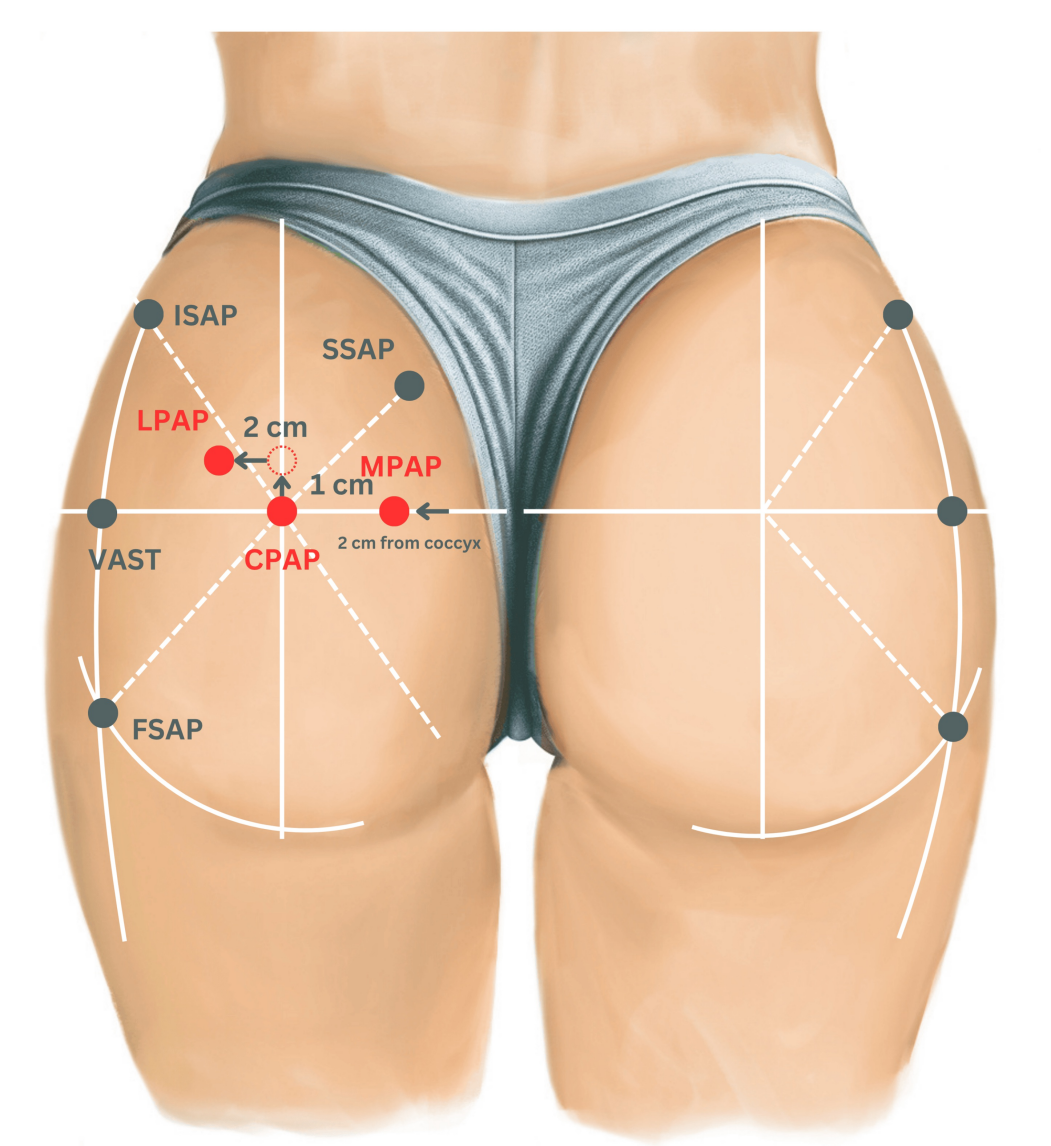
This figure was drawn by the authors to illustrate the anatomical reference points and injection landmarks used in the Round Ass Technique. ISAP, iliac secondary access point; SSAP, sacral secondary access point; LPAP, lateral primary access point; MPAP, medial primary access point; CPAP, central primary access point; VAST, vastus lateralis point; FSAP, femoral secondary access point

Finally, tertiary access points correspond anatomically to the primary and secondary ones and are used in additional treatments such as subcision and biostimulator injections aimed at fibrosis correction, collagen stimulation, and skin texture improvement (Figure 1).

Filler Injection

Once the access points are defined, proceed with volumetric correction using PMMA injection. The total amount to be injected depends on the degree of volume deficiency, as well as the patient’s height, hip and waist circumference, weight, and aesthetic expectations.

Using a 5 mL syringe and a 30 G yellow needle, inject 0.2 mL of local anesthetic at each access point to create an anesthetic bleb. Then, open the entry point with an 18 G pink needle. Using a 20 mL syringe and an 18 G/100 mm cannula, retroinject 10 mL of anesthetic solution per quadrant, ensuring the use of minimal volume to avoid compartment syndrome.

For patients shorter than 1.60 m, the primary access points are treated using an 18 G/100 mm cannula connected to syringes containing 30% PMMA. In the superomedial quadrant, inject six syringes, three through the CPAP and three through the LPAP. In the superolateral quadrant, inject another six syringes following the same distribution. The inferolateral quadrant receives nine syringes, three via each of the CPAP, LPAP, and MPAP, while the inferomedial quadrant receives six syringes, three via CPAP and three via LPAP. Injections are performed counterclockwise on the left buttock and clockwise on the right. All injections are delivered in the deep plane with 0.5 mL boluses. The deep fascia should be avoided in the medial quadrants due to the risk of neurovascular injury, while lateral injections should be subfascial. The proper vectorization of the cannula ensures even product distribution and optimal projection.

At the secondary access points, inject three syringes of 30% PMMA per site in the intramuscular plane. Retroinjections should be performed in a fanning pattern, tangential to the gluteus maximus, to maximize volumetric vectorization.

For fine adjustments, performed with the patient standing, use five syringes of 30% PMMA per access point. At the femoral secondary access point (FSAP), execute retroinjections in a fan-shaped pattern, directing 50% toward the sacral origin and 50% toward the iliac origin of the gluteus maximus. The same technique applies to the trochanteric secondary access point (TSAP). For the lateral primary access point (LPAP), inject five syringes into the deep subfascial plane, inserting the cannula at a 90° angle with up to 15° of vector deviation and using 0.5 mL boluses. This enhances the lateral border projection at the iliac origin. These refinements ensure uniform distribution and a natural aesthetic enhancement while avoiding depths that could compromise vital structures.

For subcutaneous anesthesia, perform retroinjections in a fan-shaped pattern, injecting 5 mL of the anesthetic solution per quadrant.

**Figure 2:**
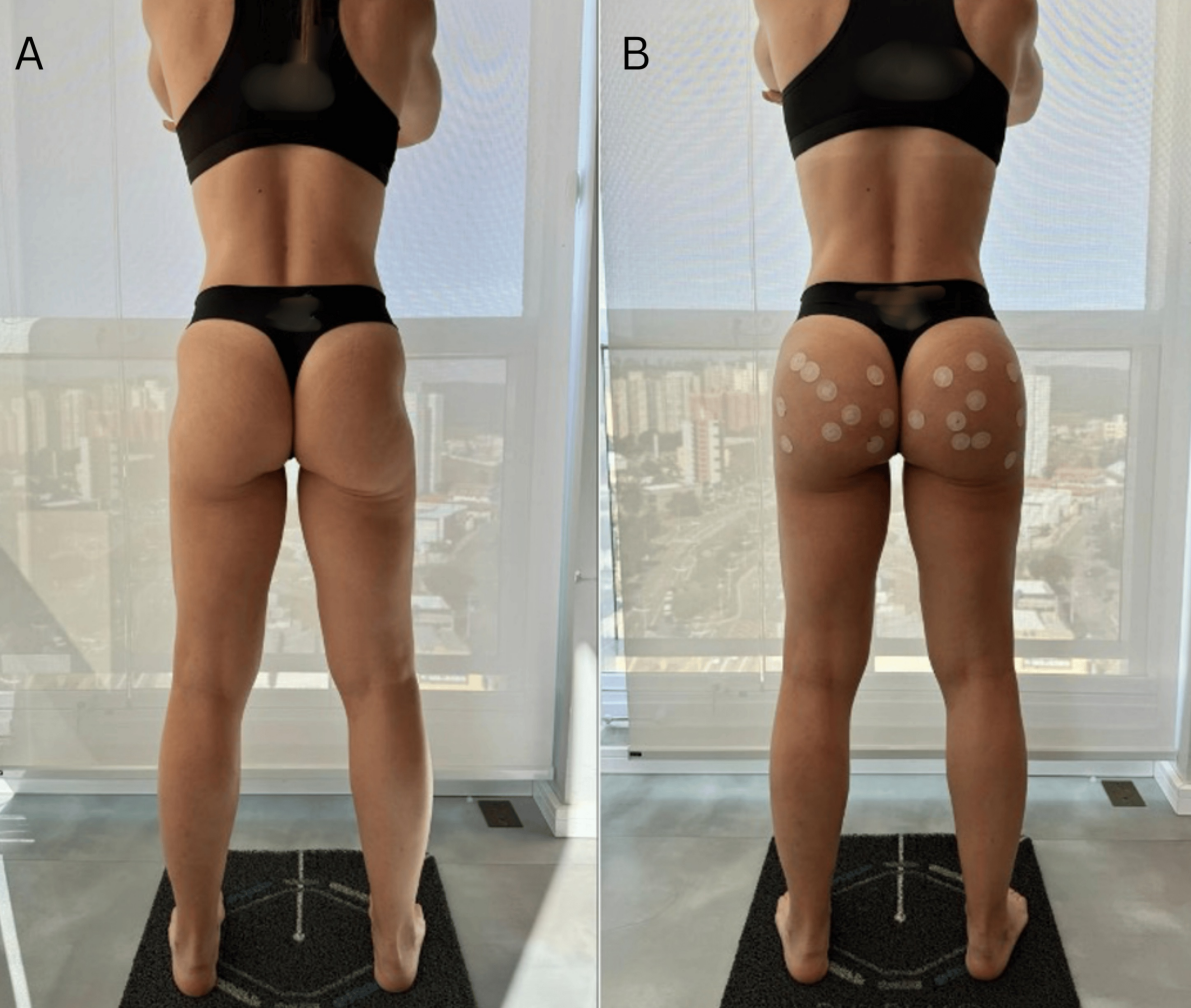
(A) Pre-procedure image showing a patient who presented with gluteal flaccidity and a pronounced hip dip. She had previously undergone multiple dermal filler and biostimulator treatments without improvement. The patient was firmly opposed to undergoing liposculpture but sought aesthetic enhancement due to significant self-esteem concerns. (B) Immediate post-procedure result following the application of our Round Ass Technique, demonstrating visible improvement in contour, projection, and symmetry.

At the tertiary access points, inject 10 syringes of 15% PMMA, five per buttock. Use a 17 G/100 mm “duckbill” cannula to perform subcision, and make minor surface adjustments as needed. With the patient in a standing position, the goal is to correct contour irregularities and achieve a more even and aesthetically pleasing gluteal surface (Figure 1). For patients between 1.60 and 1.70 m in height and those taller than 1.70 m, refer to Table 1 for a detailed summary of access point positioning and measurements. Our patient was between 1.60 and 1.70 m; for her, we followed the injection parameters presented in Table 1, and her results are demonstrated in Figure 2.

**Table 1:** Comparison of the Round Ass Technique according to patient height as determined by our team. ISAP, iliac secondary access point; SSAP, sacral secondary access point; LPAP, lateral primary access point; MPAP, medial primary access point; CPAP, central primary access point; VAST, vastus lateralis point; FSAP, femoral secondary access point; TSAP, trochanteric secondary access point; PMMA, polymethylmethacrylate; mL, milliliters; m, meters

Parameter	<1.60 m	1.60-1.70 m	>1.70 m
Primary access points used	CPAP, LPAP, and MPAP	CPAP, LPAP, and MPAP	CPAP, LPAP, and MPAP
Injection per superomedial quadrant	6 syringes (3 CPAP and 3 LPAP)	6 syringes (3 CPAP and 3 LPAP)	8 syringes (4 CPAP and 4 LPAP)
Injection per superolateral quadrant	6 syringes (3 CPAP and 3 LPAP)	6 syringes (3 CPAP and 3 LPAP)	8 syringes (4 CPAP and 4 LPAP)
Injection per inferolateral quadrant	9 syringes (3 CPAP, 3 LPAP, and 3 MPAP)	9 syringes (3 CPAP, 3 LPAP, and 3 MPAP)	12 syringes (4 CPAP, 4 LPAP, and 4 MPAP)
Injection per inferomedial quadrant	6 syringes (3 CPAP and 3 LPAP)	6 syringes (3 CPAP and 3 LPAP)	8 syringes (4 CPAP and 4 LPAP)
Secondary access points: injection per point	3 syringes of 30% PMMA each	4 syringes of 30% PMMA each	4 syringes of 30% PMMA each
Fine adjustments	5 syringes per FSAP, TSAP, and LPAP	5 syringes per FSAP, TSAP, and LPAP	5 syringes per FSAP, TSAP, and LPAP
Tertiary access (15% PMMA)	10 syringes total (5 per buttock)	10 syringes total (5 per buttock)	10 syringes total (5 per buttock)
Infragluteal fold correction (total volume)	18 mL (9 mL upper + 9 mL lower)	30 mL (15 mL upper + 15 mL lower)	42 mL (21 mL upper + 21 mL lower)
Trochanteric depression treatment (advanced technique)	30 mL of 15% PMMA (via FSAP, TSAP, and ISAP) + 30-60 mL of 30% PMMA via LPAP	Same	Same
Sacral triangle and bikini line definition	30 mL via LPAP + 30 mL via TSAP	Same	Same
Posterior projection (optional refinement)	30-45 mL via each LPAP, FSAP, and TSAP	Same	Same

## Discussion

PMMA has been used medically for over 70 years in applications such as bone cement, intraocular lenses, and vertebral support [11]. In Brazil, Biossimetric® is an ANVISA-approved PMMA filler available in concentrations from 5% to 30% [14]. PMMA induces a controlled inflammatory response, stimulating type III and I collagen formation, with microspheres remaining at the injection site, enabling long-lasting results [15]. Studies in Wistar rats confirmed PMMA’s biocompatibility, with no signs of toxicity or necrosis [16].

While PMMA may cause minor side effects such as bruising, erythema, or nodules, often due to improper injection depth, serious complications such as necrosis are rare and mostly documented in facial applications [15,16]. A 10-year cohort study of 2,770 gluteal cases reported no vascular events, likely due to the use of blunt-tip cannulas [16-18]. Risks of infection and granuloma formation are minimal with proper sterile technique and authentic products [18,19].

The granuloma rate with PMMA is estimated at 1.9%, comparable to or slightly higher than that of other fillers such as collagen and hyaluronic acid (0.1%-1%) [20]. Importantly, there were no reported cases of PMMA rejection, migration, or allergic reaction in the 10-year cohort, highlighting the product’s biocompatibility and physical stability [20].

Finally, while all injectables carry some risk, minimally invasive PMMA procedures are generally safer than surgical alternatives. With proper technique and injector experience, PMMA is a safe and effective option for gluteal enhancement [20]. In fact, a recent study showed that the use of PMMA for gluteal augmentation is safe and effective with a low incidence of adverse events (2.1%), based on a retrospective analysis of 2,801 patients and 4,725 procedures [20].

However, it is important to acknowledge that variations in preclinical techniques and patient selection criteria may influence the reproducibility of these clinical outcomes. Furthermore, while existing cohorts are substantial, there remains a notable absence of long-term, multicenter prospective data to fully validate these findings across diverse populations. Future research should prioritize the development of standardized application protocols; the inclusion of larger, multi-institutional cohorts; and extended follow-up periods to further solidify the safety profile and long-term efficacy of PMMA in large-volume augmentations.

## Conclusions

This technical report provides a definitive protocol for PMMA-based gluteal augmentation, addressing a critical gap in the literature. While the safety and permanence of PMMA are established, our team has developed this standardized, reproducible framework necessary to harness these benefits consistently. Our technique details a systematic, yet personalized, approach based on patient characteristics, facilitating predictable outcomes. We believe this standardized protocol is essential for ensuring patient safety and achieving the outstanding, permanent results that position PMMA as a safe option in gluteal aesthetics.
